# Pulse‑level characterization of low monitor unit deliveries on a modern linear accelerator using a plastic scintillation detector

**DOI:** 10.1002/acm2.70670

**Published:** 2026-06-25

**Authors:** Andrew J. White, Poonam Yadav, Indra J. Das, Ahtesham U. Khan

**Affiliations:** ^1^ Department of Radiation Oncology Northwestern Memorial Hospital, Northwestern University Feinberg School of Medicine Chicago Illinois USA; ^2^ Department of Medical Physics, School of Medicine and Public Health University of Wisconsin‐Madison Madison Wisconsin USA

**Keywords:** dosimetry, monitor unit, plastic scintillation detector, pulse‐analysis, truebeam

## Abstract

**Background:**

Variability in low monitor unit (MU) deliveries has been previously documented, but the underlying pulse‑level behavior has not been fully described due to the integrating nature of most conventional detectors. A plastic scintillation detector (PSD) enables direct visualization of MU microstructure by isolating individual linac pulses.

**Purpose:**

To use a high temporal resolution PSD to characterize pulse‑level MU substructure on a modern linac and quantify how variation in integral pulse magnitude and pulse count influence the reproducibility of low MU and fractional MU deliveries.

**Methods:**

A Blue Physics Model 11 PSD was used to measure response from individual linac pulses of a Varian TrueBeam operating at energies of 6 MV, 6 MV FFF, 10 MV, and 10 MV FFF. Initial measurements benchmarked reproducibility in pulse count and dose per pulse by delivering 100 MU at a constant dose rate of 400 MU/min. Subsequent measurements focused on low MU cases, delivering 1–3 MU at dose rates of 5–2400 MU/min.

**Results:**

The dose per pulse is variable during a single pulse train with a measured coefficient of variation (COV) of up to 13.3%. However, the average dose per pulse across multiple deliveries is stable (< 0.33% COV). This enables highly reproducible readings (< 0.23% COV) for moderate to high MU deliveries. The variation in dose per pulse coupled with a lower number of required pulses leads to challenges when delivering a low number of MUs with FFF beams. Discrepancies in the planned versus delivered MUs were observed on both detectors and the treatment delivery system.

**Conclusions:**

Time‐resolved analysis shows that dose per pulse fluctuations and the limited number of pulses comprising low MU FFF deliveries lead to measurable variability in delivered MU. These findings characterize the pulsed MU substructure on a modern linac, in which each MU is composed of a finite number of discrete radiation pulses, and quantify the achievable precision of fractional MU delivery, which is fundamentally limited by the dose per pulse (∼0.1 MU for TrueBeam FFF beams).

## 1 INTRODUCTION

Low monitor unit (MU) deliveries on clinical linear accelerators exhibit well‐documented dosimetric variability, with output inconsistencies becoming pronounced at levels below 10 MU.

Historically, linear accelerators were configured to ensure dose linearity only for deliveries exceeding 50 MU.^1^ However, modern treatment techniques often require accurate dose delivery at much lower MUs—sometimes below 10 MU. Several studies have highlighted the nonlinear dosimetric behavior of clinical accelerators at low MU levels, some even prompting the introduction of corrections to account for the observed nonlinearity.^2^, ^3^, ^4^ In contemporary modalities like IMRT and VMAT, treatment planning systems can mitigate significant dose discrepancies by establishing a minimum MU threshold per segment.^5^, ^6^, ^7^, ^8^

With previous generation linac models, the increased uncertainty at low MU levels was attributed to the suboptimal sampling frequency and communication between control systems.^7^, ^9^ As linacs evolved and electronics within the controllers got faster, the overshoot effect became less of a concern.^7^, ^10^ While these improvements mitigated overshoot in flattened beams, the introduction of flattening filter free (FFF) configurations in the early 2010s—with higher dose rates and dose per pulse—reintroduced beam control challenges. One of the main topics explored in this study was to determine how a higher dose per pulse beam impacts the ability to precisely deliver a fractional MU.

Despite previous observations, the underlying mechanisms, particularly the characteristics of the pulse train contributing to an MU, remain underexplored. One contributing factor is the temporal resolution limitations of conventional external dosimetry systems, which integrate charge over the entire delivery and lack the ability to monitor pulse‐to‐pulse fluctuations. Additionally, the signal from each linac pulse is too low to provide a sufficient signal to noise ratio with traditional thimble ion chamber detectors.

Plastic scintillation detectors (PSDs) offer a promising solution to these issues, providing a very high signal to noise and sub‐millisecond temporal resolution that is capable of isolating discrete pulse events. By capturing the integrated magnitude of individual linac pulses, this study aims to enable detailed analysis of dose per pulse variability and pulse count stability using a commercially available PSD.

## 2 METHODS AND MATERIALS

### 2.1 Detectors

A Blue Physics Model 11 PSD (Tampa, FL) was used for this study due to its desirable sampling interval (500 µs), which allows for the isolation and integration of discrete linac pulses.^11^ The detector consists of a 1 mm diameter by 1 mm length cylindrical scintillator that is coupled to an optical fiber which transports light signal to the acquisition unit. A second fiber (without a coupled scintillator) runs adjacent in the cable to facilitate Cherenkov subtraction during signal processing. The acquisition unit and fiber optic cartridge are positioned in the maze of the vault during measurements to minimize radiation‐induced damage to the photomultiplier tube (PMT) and analog‐to‐digital converter (ADC). Finally, the acquisition unit is connected via data cable to a laptop outside the vault to allow for real‐time (active) dosimetry. Blue Physics provides its own commercial software (BlueSoft) to collect and analyze data. The software allows users to select between two sensitivity settings, designated by the rank (capacitance) of the capacitor utilized in the acquisition unit. The highest sensitivity (Rank 0–10 pF) was used throughout this study without saturating the capacitor. A more complete description of the Blue Physics PSD system, including a detailed discussion on Cherenkov subtraction formalism, can be found in Das et al^11^.

A PTW 30013 (Freiburg, Germany) farmer ion chamber was also placed in the beamline to serve as a time‐integrated reference signal to the integrated PSD signal. Integrated charge was read out using a PTW Unidos Romeo electrometer with a 10 fC resolution. The main goal of this study was to analyze linac pulse variability, enabling relative measurements and therefore no calibration coefficient was applied.

### 2.2 Experimental setup

A Varian TrueBeam v3.0 (Palo Alto, California) was used for all irradiations. Photon energies of 6 MV, 6 MV FFF, 10 MV, and 10 MV FFF were investigated with dose rates ranging from 5 to 2400 MU/min. Exact dose rates were selected on an energy‐specific basis but included the highest and lowest clinical dose rates for the respective energy, plus a third dose rate close to the median.

**FIGURE 1:**
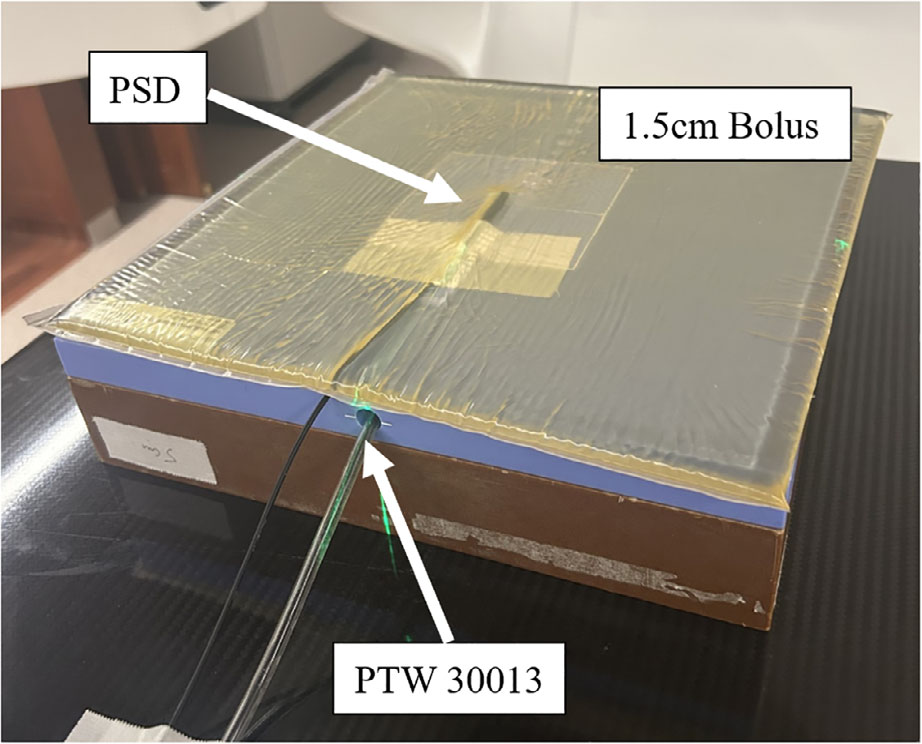
Experimental setup. A Blue Physics PSD and PTW 30013 farmer ionization chamber were positioned in a 10 × 10 cm^2^ beam at 100 cm SSD. The PSD was placed beneath 1.5 cm bolus, while the ion chamber was embedded in water‐equivalent material with 5 cm backscatter.

Figure 1 illustrates the measurement setup. The ion chamber was placed inside a 2 cm slab of water‐equivalent plastic, which was stacked atop an additional 5 cm of water‐equivalent material for backscatter. The PSD was centered on the linac crosshairs and placed under 1.5 cm of flexible 30 × 30 cm^2^ bolus. The source to surface distance (SSD) and field size were set to 100 cm and 10 × 10 cm^2^, respectively.

Prior to examining the impacts of low MU deliveries, the variability in number of pulses and dose per pulse was assessed on more conventional MU deliveries. For these preliminary measurements, 100 MUs were delivered at a consistent dose rate of 400 MU/min (highest clinical dose rate available across all photon configurations investigated). Three trials were conducted for each energy.

For investigation of low MU deliveries, five trials of 1, 2, and 3 MUs were measured for each energy and dose rate (3 MU values x 5 trials x 4 energies x 3 dose rates = 180 total measurements). Even as modern linacs have advanced past integer MU requirements, 1 MU remains the lower bound on the TrueBeam and most clinical accelerators. In addition to saving the raw PSD pulse data and ion chamber measurements, the delivered MUs were also recorded from the TrueBeam Treatment Delivery System (TDS).

Field size dependence was also characterized to assess potential beam steering variations during the startup phase. Utilizing the same measurement geometry as the primary dataset, irradiations were performed with square field sizes of 1, 2, 3, and 10 cm^2^. Due to the lower signal associated with small fields, ten trials were conducted for each field size and MU (1, 2, 3, 100) combination.

To account for potential gravitational effects on beam startup characteristics, the angular dependence of low MU deliveries was also investigated. Measurements were performed at the four cardinal gantry angles (0°, 90°, 180°, and 270°) with the PSD suspended in air and fitted with a 0.5 cm buildup cap. For each angle, five trials were recorded for settings of 1, 2, and 3 MUs.

The field size and gantry angle irradiations were both configured with a 10 MV FFF beam operating at 2400 MU/min.

### 2.3 Pulse analysis

Analyzing data with the commercial BlueSoft software is sufficient for most clinical applications. However, due to the large amount of measured data and the desire to extract additional statistical information from the pulse trains, an in‐house MATLAB script was developed to perform the analysis. The Blue Physics PSD system generates a comma‐separated values (csv) file after each measurement, which stores the values of time, scintillator voltage, and Cherenkov voltage required to complete the analysis. To obtain the scintillator signal that is proportional to dose, the adjacent channel ratio (ACR) must be applied to correct the scintillator readings for Cherenkov. Multiple methods exist for determining the ACR for the PSD system as described in several references.^11^, ^12^, ^13^ This work utilized the collimator rotation method as outlined by Khalifa et al.—a rectangular field size of 3 cm x 6 cm rotated through 90° in 10° increments.^13^

A sampling interval of 500 µs was used for this study, which corresponds to the rate at which the capacitor (voltage) is readout in the acquisition unit. Given the nominal pulse width of ∼3 µs and a scintillation decay time of 5 ns, the PSD is well‐suited to discretize individual pulses.^14^ If the linac pulse is delivered simultaneously with the capacitor readout, a second capacitor is switched into the circuit ensuring a negligible loss of signal. At 500 µs, the PSD sampling interval is approximately 5.6 times faster than the pulse repetition frequency of the TrueBeam (360 Hz ∼ 2780 µs between pulses at the highest clinical dose rates).^15^ Therefore, the signal from sequential readouts can be combined to form a single pulse of the full height. This design feature must be accounted for to avoid double counting pulses during analysis.

**FIGURE 2:**
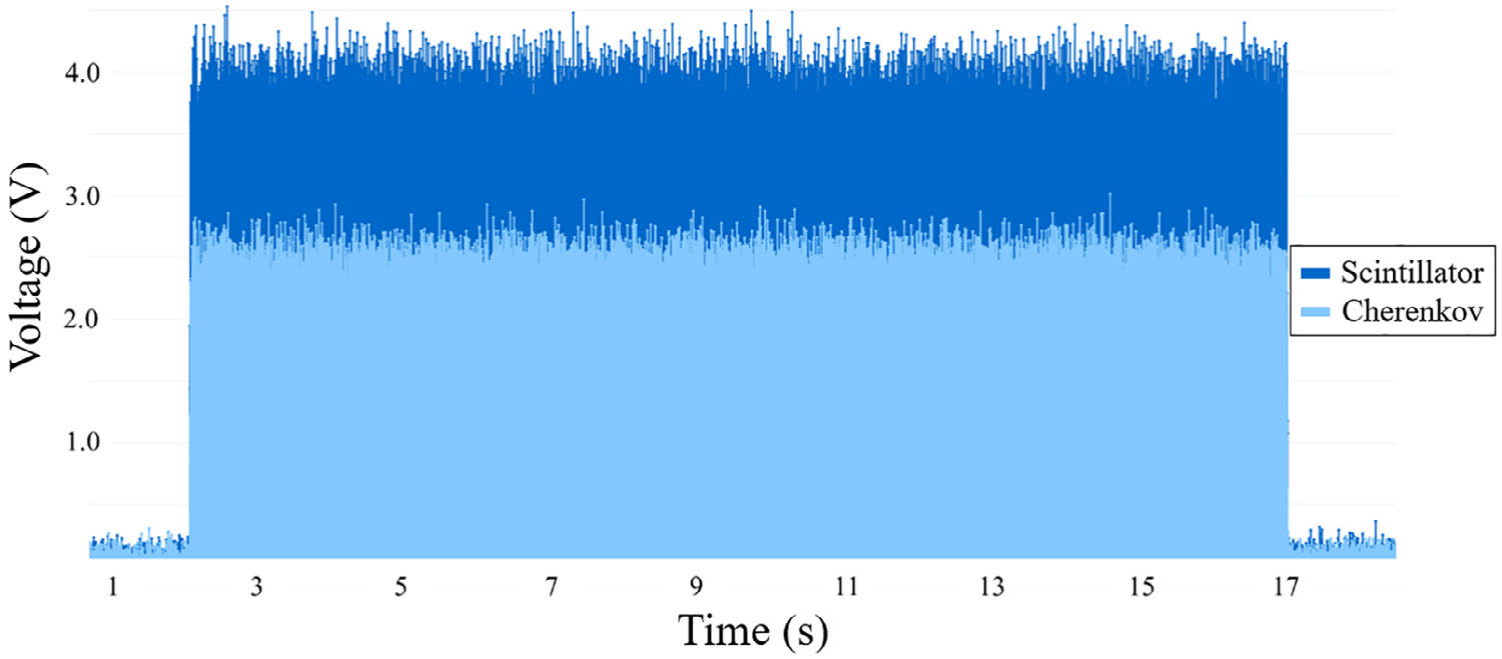
Raw data for 100 MU, 6 MV, 400 MU/min pulse sequence. Each time interval includes two readouts: the total scintillator signal from the detector and a Cherenkov signal from the adjacent channel. The experimentally determined ACR is applied to the scintillator signal to yield the charge proportional to dose. An in‐house MATLAB script was written and validated against the commercial software to extract additional pulse statistics.

The script used to analyze the data in this study was validated against the commercial BlueSoft software before being implemented. An example of the raw pulse data visualized through the BlueSoft interface is displayed in Figure 2.

## 3 RESULTS

### 3.1 Conventional MU deliveries

Table 1 provides a summary of the ion chamber and pulse statistics for the 100 MU trials. As expected, the reproducibility in dose between the three trials, quantified by the coefficient of variation (COV), was extremely high across detector type and energy (< 0.23% COV).

**TABLE 1:** Ion chamber readings and pulse statistics for 100 MU deliveries across all investigated beam energies at 400 MU/min. The coefficient of variation (COV) was used to quantify reproducibility within the variables.

	Ion chamber	Blue physics PSD				
Energy	Dose COV (%)	Dose COV (%)	Avg dose per pulse COV ‐ 3 trials (%)	# of pulses COV (%)	Avg # of pulses	Dose per pulse COV—single trial (%)
**6 MV**	0.00	0.11	0.23	0.17	3548	13.30
**6 MV FFF**	0.06	0.23	0.33	0.15	1384	6.60
**10 MV**	0.00	0.13	0.26	0.13	3436	12.13
**10 MV FFF**	0.06	0.13	0.00	0.15	792	4.33

For the PSD measurements, the average dose per pulse was calculated for each trial. There was very little observed variation (< 0.33% COV) in the averages between trials. However, there was appreciable variation (up to 13.3% COV) in dose per pulse within individual pulse trains, with the largest relative fluctuations occurring in lower dose per pulse beams (6 MV and 10 MV). As illustrated in Figure 2, 100 MU deliveries require a vast number of individual pulses—over 3400 pulses for flattened beams. While the total number of delivered pulses was high, the variation in pulse count was minimal across all energies (< 0.17% COV).

### 3.2 Low MU deliveries

**FIGURE 3:**
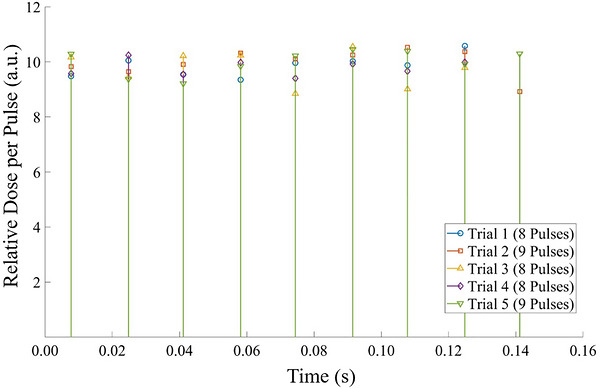
Pulse sequences for 1 MU, 10 MV FFF, 400 MU/min trials. There is appreciable variation in dose per pulse throughout the delivery and an additional pulse is observed in two of the trials. The same data is also presented in Table 2, along with data from the other detectors. a.u.: arbitrary units derived from the scintillator signal corrected for Cherenkov.

As discussed in Section 3.1, the variation in dose per pulse is lower for 10 MV FFF beams. However, the high dose per pulse means that fewer pulses are required to compose an MU. Figure 3 and Table 2 show that each MU is subdivided into approximately eight pulses for 10 MV FFF beams. In some trials, consistently higher integrated readings on the ion chamber and PSD coincided with an additional pulse being recorded by the PSD. The Varian TDS also recorded delivered MU values that exceeded the planned value (e.g., 1.1 MU for a 1.0 MU plan), consistent with the additional pulse identified by the PSD. This behavior was not limited to 1 MU deliveries, as overshoots of 2.1 and 3.1 MU were also observed at other MU levels (see Table 2). Additionally, the overshoot effect was observed in numerous trials across all dose rates. The repeated presence of these discrepancies across all investigated MU levels and dose rates indicates that the precision of fractional MU deliveries for FFF beams is primarily constrained by the discrete number of pulses composing each MU, with variations dominated by the inclusion of an additional pulse rather than changes in individual pulse height.

**TABLE 2:** Example of the overshoot effect being observed across detector types and at all low MU levels.

	TrueBeam TDS		Ion chamber	Blue physics PSD		
Trial	Planned MU	Delivered MU	Charge (pC)	Voltage (V)	# of Pulses	Figure 3
1	1.0	1.0	179.0	78.9	8	X
*2*	*1.0*	*1.1*	*202.0*	*89.9*	*9*	*X*
3	1.0	1.0	179.5	78.3	8	X
4	1.0	1.0	179.5	78.3	8	X
*5*	*1.0*	*1.1*	*201.5*	*90.0*	*9*	*X*
1	2.0	2.0	360.5	162.4	16	
*2*	*2.0*	*2.1*	*381.5*	*167.0*	*17*	
*3*	*2.0*	*2.1*	*383.5*	*170.6*	*17*	
*4*	*2.0*	*2.1*	*382.0*	*168.6*	*17*	
5	2.0	2.0	360.5	160.3	16	
1	3.0	3.1	565.0	250.7	25	
2	3.0	3.0	541.5	240.7	24	
*3*	*3.0*	*3.1*	*563.0*	*247.4*	*25*	
4	3.0	3.0	541.5	242.8	24	
*5*	*3.0*	*3.1*	*564.5*	*248.5*	*25*	

*Note*: In trials with an additional pulse delivered, a proportional increase in signal is observed in the integrated readings. For continuity, the first 5 rows of data presented in this table (10 MV FFF, 400 MU/min, 1 MU) match the pulse trains displayed in Figure 3. Trials with MU discrepancies are italicized to emphasize the overshoot effect.

### 3.3 Dependence on field size and gantry angle

**FIGURE 4:**
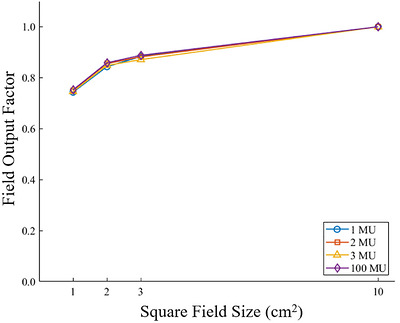
Effect of field size. Field output factor (FOF) as a function of square field size for various MU deliveries (10FFF, 2400 MU/min). The difference in FOFs was found to be negligible (COV < 0.82%).

**FIGURE 5:**
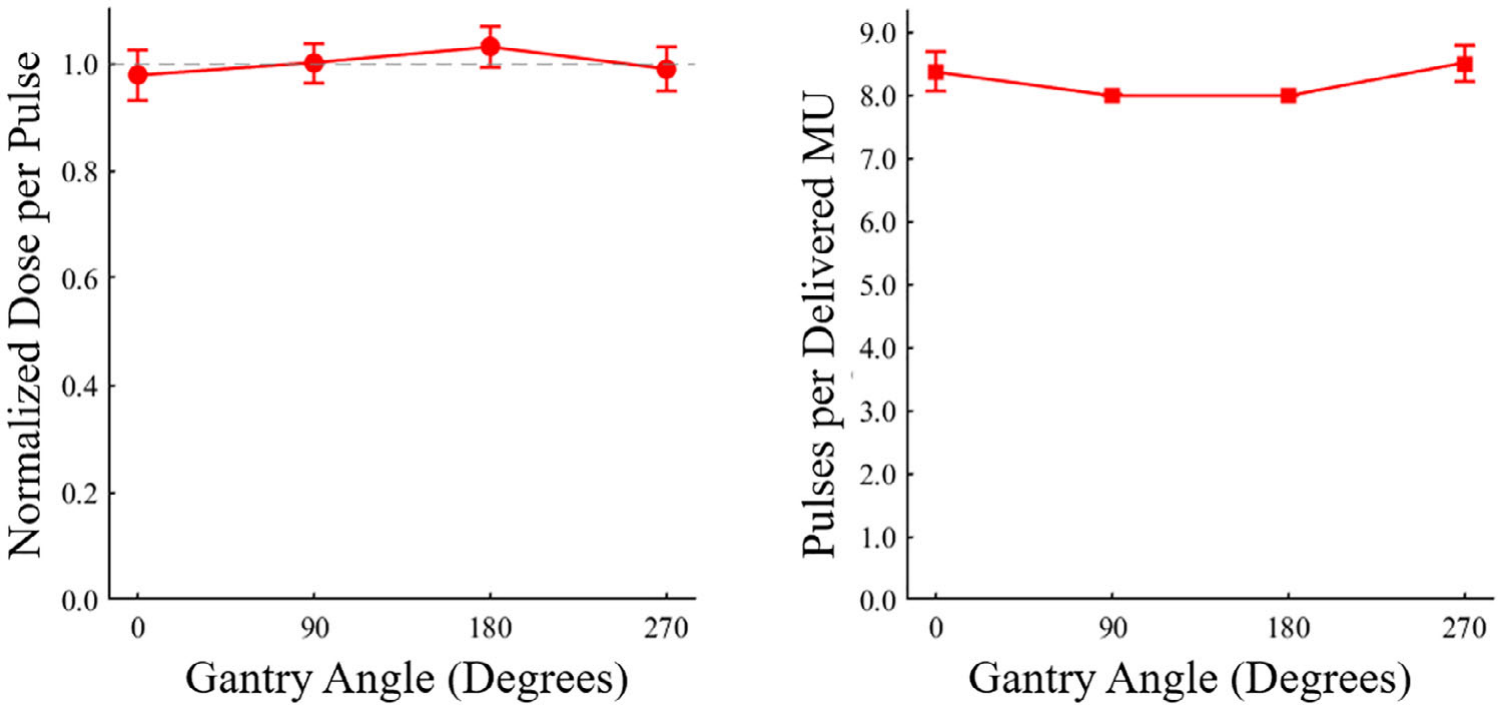
Effect of gantry angle. Normalized dose per pulse (left) and pulses per delivered MU (right) as a function of gantry angle measured in air with 0.5 cm buildup cap. Dose per pulse was normalized to the average of the four cardinal angles to minimize uncertainty due to detector alignment. Error bars represent the standard deviations across the 15 trials at each gantry angle (1, 2, and 3 MUs combined).

As shown in Figure 4, the field output factors (FOFs) derived from 1, 2, and 3 MU deliveries did not deviate from the 100 MU baseline (COV < 0.82%). This stability is expected, as the transient beam startup occurs upstream of the collimating jaws. Furthermore, measurements performed at cardinal gantry angles (Figure 5) revealed that the linac's startup behavior is independent of gravitational orientation, with no significant variation observed in either the relative dose per pulse or number of pulses delivered per MU. To eliminate the effects of the overshoot discussed in Section 3.2, all analyses were normalized to the exact MUs delivered as recorded by the TDS (e.g., utilizing 3.1 MU rather than the planned 3.0 MU). Ultimately, the overshoot was observed in every mechanical configuration investigated and the frequency of occurrence was found to be independent of both gantry angle and field size.

## 4 DISCUSSION

This study provides unique visualization and quantification of the MU microstructure on a commercial linac. The data shows appreciable variability in pulse height for 6 MV and 10 MV beams, with less relative variability observed in FFF beams. The impact of this variation for flattened beams is diminished by the large number of pulses delivered during treatments that contain many MUs. As seen in Table 1, a 100 MU delivery consists of over 3400 pulses for both flattened beams.

**FIGURE 6:**
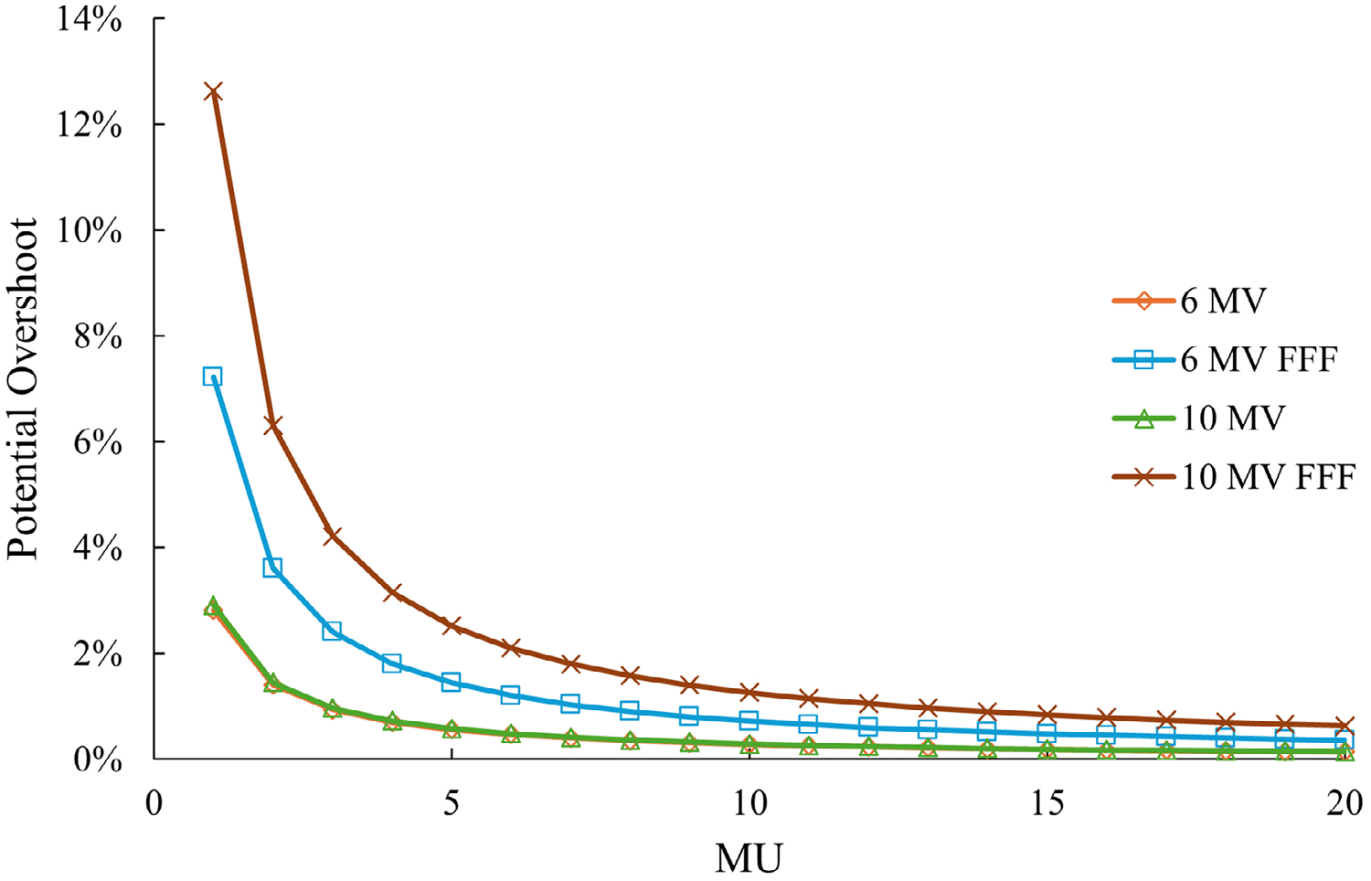
Analytical solution to pulse overshoot. The theoretical dosimetric impact of the linac delivering one additional pulse is plotted as a function of total MU. Higher dose per pulse beams (FFF) have a larger potential overshoot due to the fewer number of pulses per MU. Note that due to the similar number of pulses per MU, the 6 MV and 10 MV curves overlap.

Figure 3 and Table 2 show an example of 10 MV FFF trials where an extra pulse was delivered and contributed to an overresponse on all detectors investigated and the TrueBeam TDS. Figure 6 further develops this idea by plotting the theoretical dosimetric consequence of delivering one additional pulse for each energy as a function of total MU. This analytical solution agrees with the magnitude of overresponse that was measured in this study, as well as what has been previously reported in literature.^6^, ^7^, ^9^ The clinical significance of this overshoot is minimal, as even for the highest dose per pulse beam (10 MV FFF), the dose contributed by an additional pulse is < 2 mGy.

Our measured overresponse of the 10 MV FFF beam aligns with what has been previously published by Li et al.^7^ Their work reported a 10 percent dose difference for 10 MV FFF beams of 1 MU on a TrueBeam operating at 2400 MU/min.

While this study utilized external detectors to characterize the pulse stability of the linac, the TrueBeam beam generation system (BGM) is also capable of monitoring the dose per pulse by operating the monitor chamber at a high voltage (500 V) that quickly separates the liberated ions within the respective air cavities.^16^ According to the Varian TrueBeam Technical Reference Guide, the BGM terminates the beam prior to the next pulse if one of the following criteria are exceeded:

- The dose per pulse is 30% greater than the expected dose per pulse, for any single pulse
- The dose per pulse is 50% less than the expected dose per pulse, for five consecutive beam pulses, starting after the first 100 beam pulses

These rather loose criteria highlight the fact that variation in pulse height, or even pulse dropout, is to be expected on the TrueBeam during treatment.

**FIGURE 7:**
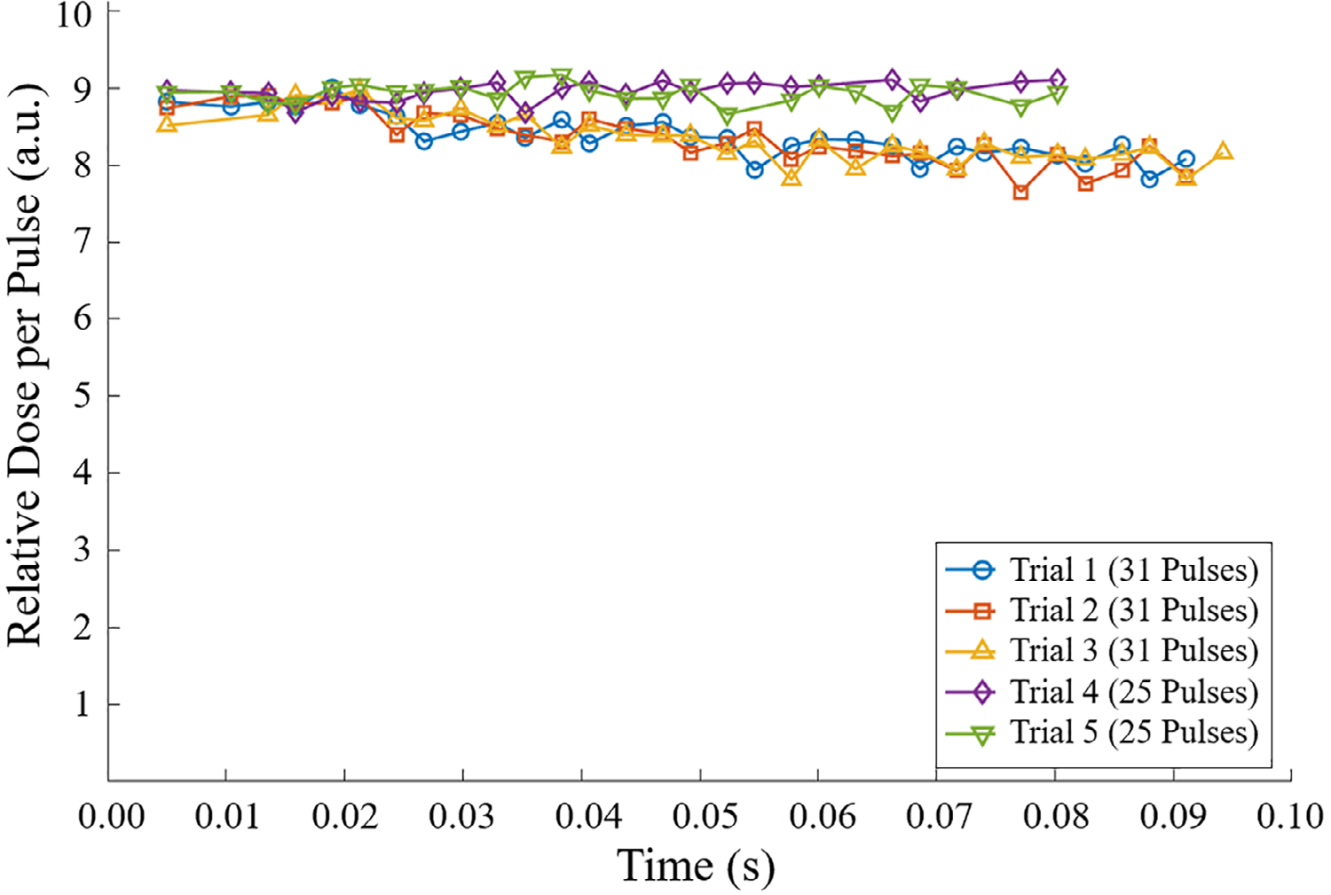
Pulse sequences for 3 MU, 10 MV FFF, 2400 MU/min, 2 × 2 cm^2^ field size trials. Pulse heights were connected to help identify trends throughout the delivery and amongst the different trials. It is observed that trials 4 and 5 had a higher relative dose per pulse during the delivery. The monitor chamber accounts for this deviation by terminating the beam sooner (in this case delivering 6 fewer pulses compared to trials 1, 2, and 3). a.u.: arbitrary units derived from the scintillator signal corrected for Cherenkov.

Figure 7 shows both variation in dose per pulse and the number of pulses delivered across multiple trials. This example (3 MU, 10 MV FFF, 2400 MU/min, 2 × 2 cm2 field size) was selected to illustrate the interplay between these two variables and how they influence the active feedback loop with the monitor chamber. Trials 4 and 5 have a higher average dose per pulse, therefore the monitor chamber terminated the beam following 25 pulses. However, in trials 1, 2, and 3 the dose per pulse decreased throughout the delivery. To compensate for this lower output, the linac delivered 6 additional pulses (total of 31) before the monitor chamber terminated the beam. No delivery settings were intentionally varied between trials; therefore, the observed differences in dose per pulse reflect intrinsic short‐term fluctuations in linac output rather than controlled changes in delivery parameters. Pulse dropout can also be visualized in Trial 3 (orange) of Figure 7. The second data point is missing from this pulse train but is compensated for by delivering one pulse beyond the other trials with 31 total pulses delivered.

Future work may include investigating other linac vendors, as beam generation and modification are approached differently amongst vendors.^17^ Additionally, it may be useful to repeat the study with electron beams. As previously mentioned, the impact of the current study is limited due to the low dose per pulse (< 2 mGy) from clinical photon beams. However, as research groups begin to explore the conversion of clinical linacs into FLASH‐capable machines (dose per pulse ∼1 Gy), it becomes even more critical to understand the beam controlling mechanisms and their limitations.^18^ Preliminary studies like this may contribute to the design of the next generation of clinical linacs that will be used for FLASH treatment.

## 5 CONCLUSION

This study provides insights into the pulse variability that contributes to poor reproducibility during low MU deliveries by examining the microstructure of the MU, using a commercial PSD to analyze individual linear accelerator pulses. It was determined that a combination of variability in the dose per pulse and the varying number of pulses that make up an MU contribute to the overshoot effect observed in many of the 10 MV FFF trials. This work also highlights the level of precision that is achievable when delivering fractional MUs with a high dose per pulse beam (10 MV FFF) on a modern TrueBeam.

## AUTHOR CONTRIBUTIONS

Andrew J. White performed measurements, completed the scientific analysis, and wrote the manuscript. Ahtesham U. Khan conceived the research idea and performed measurements. Indra J. Das and Poonam Yadav contributed to the research idea and supervised the project. All authors discussed the results and edited the final manuscript.

## CONFLICT OF INTEREST STATEMENT

The authors declare no conflicts of interest.

## Data Availability

Research data are stored in an institutional repository and will be shared upon request to the corresponding author.
